# Consensus on disease control objectives in the context of COVID-19 vaccines

**DOI:** 10.2471/BLT.20.283846

**Published:** 2021-05-01

**Authors:** Vageesh Jain, Sam Tweed

**Affiliations:** a Institute for Global Health, University College London, 30 Guilford Street, London WC1N 1EH, England.

Most governments are pursuing control strategies to reduce coronavirus disease 2019 (COVID-19) incidence and mortality. Unlike targets for global or regional disease elimination that tend to be set to a uniform standard by the World Health Organization (WHO), metrics for COVID-19 control are determined at a national level. However, the roll-out of COVID-19 vaccines warrants a clear and shared international understanding of disease control.

Global health actors have committed to equitable global access to innovative tools for COVID-19 for all, through the Access to COVID-19 Tools (ACT) Accelerator, of which the COVAX Facility is one of three pillars.^1^ However, to integrate vertical equity – that is, prioritizing those most in need – into global COVID-19 control efforts, countries must be able to refer to a normative standard against which various existing epidemiological metrics can be benchmarked. Because global vaccine distribution is uneven,^2^ without this standard some countries will meet their locally determined targets, while others with similar needs will not. A single international classification will enable a more objective and standardized assessment of both vaccine needs and disease control across countries. Once national epidemics come under sufficient control as measured against such an international standard, advancing discussions on equity, strategic aims and global cooperation will be possible.

Under the current COVAX Facility system for global vaccine allocation, countries receive vaccine doses proportional to the size of their population to cover up to 20% of citizens.^3^ Once this threshold has been reached, countries receive doses based on an assessment to estimate need. The proposed parameters to assess need include effective reproduction number and its trend, hemisphere location, universal health coverage service index, health system saturation and high-risk groups for COVID-19. Each country will be given a risk score based on the weighted averages of these parameters, accompanied by a qualitative assessment to account for country context. The current approach provides a relative measure of vaccine needs across countries that is valuable for decision-makers. However, without a shared and explicit understanding of disease control, national leaders will continue to pursue objectives in line with their perceived needs.

Clearly defining disease control will build on existing WHO plans. To estimate the extent of disease control in a country or region, routine and internationally comparable metrics must be selected. In addition, the range of thresholds at which epidemics can be considered poorly, adequately or well controlled must be determined. An analysis of trends in case numbers across whole populations or stratified by age, positivity rates, health system pressures and other indicators might be necessary. Combining this analysis with real-time intelligence on local vaccine supply will help to identify areas where focused public health efforts may be required to increase the uptake of vaccines to better control disease. 

As more vaccines become available, outbreaks come under control in some areas and global cooperation becomes more of a priority, a disease control classification for COVID-19 will be a key resource to benchmark vaccine needs across countries. Countries with a high level of disease control may be more willing to engage with equitable global vaccine allocation efforts, and may increasingly be compelled by their citizens to do so. If elimination or eradication is not the goal, the added benefit of securing more vaccine doses may be negligible and the opportunity costs for global disease control significant. 

For pandemic influenza, the existing World Health Assembly legislative framework mandates that WHO distributes vaccines to countries on the basis of public health risk and needs.^4^ For COVID-19, such risks and needs are likely to change over time. If wealthy countries choose to go beyond disease control in a vaccine-scarce world, pursuing elimination or giving booster doses as proposed in the United Kingdom of Great Britain and Northern Ireland,^5^ then poorer countries may be disadvantaged. Until varying disease control aims can be made explicit via reference to a common definition, whether and when countries make this transition will be largely unknown, and down to local rather than global priorities.

Despite welcome initiatives such as the COVAX Facility, estimating needs for COVID-19 vaccines across countries is difficult. Using vaccines to pursue locally determined disease control targets will lead to a fragmented and inequitable global pandemic response. Specifying the epidemiological parameters at which COVID-19 outbreaks can be considered well controlled under various vaccination scenarios is essential. As population immunity changes heterogeneously around the world, a single international classification will inform our understanding of national needs for vaccines, diagnostics, therapeutics and other public health interventions.
